# A Case of Visceral Leishmaniasis in a 4-Year-Old Child Living in Nonendemic Area Located in Suburbs of Dakar, Senegal

**DOI:** 10.1155/2023/2354935

**Published:** 2023-09-09

**Authors:** Magatte Ndiaye, Dienaba Fafa Cissé, Aicha Djigal, Aminata Sow, Souléye Lélo, Fatoumata Ly, Isaac A. Manga, Mame Ami Diouf, Doudou Sow, Oumar Gaye, Boubacar Camara, Babacar Faye

**Affiliations:** ^1^Parasitology-Mycology Department, Faculty of Medicine University Cheikh Anta Diop, Dakar, Senegal; ^2^Laboratory Diagnostic, Pikine Teaching National Hospital, Pikine, Senegal; ^3^Paediatric Department, Pikine Teaching National Hospital, Pikine, Senegal

## Abstract

Visceral leishmaniasis (VL) is an infectious disease caused by protozoa of the genus *Leishmania*. Sporadic cases are observed in nonendemic areas and often associated with limited foci; therefore, the disease is easily overlooked. In addition, other diseases have similar clinical symptoms, which make it difficult for clinicians to make an accurate diagnosis and to provide effective treatment. We identified visceral leishmaniasis in a 4-year-old child in Pikine, Senegal. The patient was admitted to the Pikine National Teaching Hospital for haemorrhagic, tumoral, and infectious syndromes. At admission, the patient presented with epistaxis and gingivorrhagia, a severe anaemic syndrome poorly tolerated, a systemic inflammatory response syndrome with fever at 39.5°C, a tumoral syndrome with 11 cm of hepatomegaly and 12 cm of type IV splenomegaly, and noninflammatory macropoly adenopathies. A spinal cord puncture was performed, and direct microscopy examination of the sample after GIEMSA staining revealed amastigote forms of *Leishmania*. The PCR amplification of extracted DNA from the bone marrow aspiration using specific primers for VL (forward and reverse) confirmed that VL was responsible for the infection. A treatment with meglumine antimoniate (Glucantime) was given and it gave a successful outcome with remission of clinical symptoms and favourable evolution with 3 months hindsight. *Conclusion*. This visceral leishmaniasis case diagnosis in Senegal has shown that, apart from haematological malignancies, this disease must be considered in combination with a tumor syndrome, haemorrhagic syndrome, and infectious syndrome.

## 1. Introduction

Leishmaniasis is a vector-borne disease with a complex ecology and epidemiology. In humans, they are caused by more than 20 species of the genus *Leishmania* living in different types of ecosystems and causing various clinical manifestations (mainly cutaneous, mucosal, or visceral) [1]. In Senegal, studies have shown that in areas where human leishmaniasis is endemic, cutaneous forms due to *L. major* were mainly observed [1, 2]. However, in the rural community of Mont-Rolland, *L*. *infantum* has been identified since 1970 as the cause of canine leishmaniasis [1, 2]. More recent studies have shown that the circulation of *L. infantum* was well established in this past outbreak and that more than 30% of dogs and 20% of humans were serologically positive [3, 4]. Furthermore, authors have shown the presence of *L. infantum* in cutaneous leishmaniasis in a child with HIV [5]. Thus, several factors make Senegalese leishmaniasis an atypical and worrisome focus: (1) an unusual location of *L. infantum*; (2) the hyperendemicity of canine leishmaniases in the Thies region; and (3) a potential risk for human health.

In this epidemiological context favourable to the transmission of *L. infantum* in humans, in addition to the clinical arguments, it was important to set up a molecular diagnosis platform for VL in addition to the standard methods and clinical symptoms of VL.

## 2. Study Population

We reported an observation of a 4-year-old boy, without any particular perinatal history, with a good psychomotor development and who have received all immunizations according to Senegal's Expanded Programme on Immunization (EPI).

He was a son of 29-year-old mother, second gesture, second pare, and a 35-year-old father, both parents were without any medical surgical antecedents or family defects. He is the oldest of two siblings, the second is 13-month-old and in good health.

## 3. Study Area

The patient lives in Thiaroye district, in the suburbs of Dakar, characterized by insalubrity with stagnant water, swampy areas, and domestic animals roaming around. This particular biotope is favourable for the circulation of the *Phlebotomus* insects, vectors of *Leishmania* species. In the household, all family members slept under impregnated mosquito nets, and prior to admission, the patient had not travelled outside the residence area.

## 4. Description of Para-Clinical Parameters

He was admitted to the Paediatric Department of the Pikine National Teaching Hospital on November 25^th^, 2019, for a worsening of epistaxis and gingivorrhagia that had been manifesting sporadically for 3 months without any real follow-up.

At admission, the patient had fever with a body temperature at 39.5°C, a heart rate of 153 btt/min, respiratory rate of 48 c/min, and capillary blood glucose of 1.3 g/L. Blood pressure was normal at 93/62 mmHg, and arterial oxygen saturation was 97%. Weight and height were 12 kg and 97 cm, respectively, with a ratio of weight/height at −2 SD and brachial perimeter at 13 cm.

## 5. Clinical Diagnostic

Clinical examination revealed haemorrhagic syndrome with bilateral epistaxis of great abundance with clotting, severe anaemic syndrome that was poorly tolerated, moderate respiratory distress syndrome, and systemic inflammatory response syndrome with fever at 39.5°C. A tumor syndrome with hepatomegaly of 11 cm, 12 cm splenomegaly type IV of the Hackett classification, and noninflammatory macropoly adenopathies in the cervical and axillary areas were noted. In addition, there were multiple mouth ulcers. Examination of the other organs was unremarkable. A chest X-ray had revealed bilateral pneumonia, and abdominal ultrasound, apart from objectified hepatosplenomegaly, was normal.

The diagnosis of malignant haemopathy was evoked with blood count which showed hypochromic microcytic anaemia (3.44%/79500) with a haemoglobin level at 05.1 g/dl and severe thrombocytopenia at 3000/mm^3^. However, the smear was not in concordance with level of 7% blasts and 3% erythroblasts.

Faced with this infectious syndrome, we looked for and eliminated malaria, retrovirosis, hepatitis, and severe sepsis picture in a particular field such as sickle cell disease or vitamin B9 or B12 deficiency.

Thus, the diagnosis of visceral leishmaniasis was evoked based on clinical symptoms such as tumor syndrome with splenomegaly and adenopathy. We collected a lymph node biopsy for microscopy examination; however, the results were negative. As other probable conditions were formally ruled out, bone marrow aspiration was performed for diagnosis.

## 6. Biological Diagnostic of Leishmaniasis

### 6.1. Microscopy Examination

**Figure 1:**
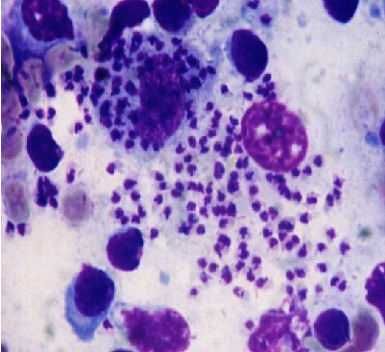
Microscopy examination of amastigote form extracellularly and intracellularly (100×).

The biological sample was sent to the Parasitology-Mycology Reference Laboratory at the Faculty of Medicine, Cheikh Anta Diop University of Dakar, for microscopy and molecular diagnostic. For microscopy identification, direct examination of the spinal cord cytopic fluids after GIEMSA staining revealed amastigote forms of *Leishmania* (Figure 1).

### 6.2. Molecular Diagnosis

For molecular diagnostic, parasite DNA was extracted from collected blood samples by QIAmp DNA blood mini kits, following the manufacturers' protocol. Extracted DNA was amplified in 96-well PCR microplates by the CFX96 qPCR machine. For the outer PCR primers, CSB2XF (C/GA/GTA/GCAGAAAC/TCCCGTTCA) and CSB1XR (ATTTTTCG/CGA/TTTT/CGCAGAACG) were used. The 30 *µ*L *of* outer PCR mixture consisted of the CSB2XF/CSB1XR (1 *µ*M/primer) and 1.0X TEMPase Hot Start Master Mix (including 3.0 mM MgCl_2_, 0.4 mM dNTPs, and 0.2 units/*µ*l TEMPase Hot Start DNA Polymerase) and 1 *µ*L of extracted DNA. The reaction mixture of the nested PCR was identical to that of the outer PCR, and the primer sets 13Z (ACTGGGGGTTGGTGTAA AATAG) [6] and LiR (TCGCAGAACGCCCCT) [7] were used. Positive and negative controls were included during the PCR. The nested PCR products were confirmed by running the controls by electrophoresis on a 1.5% agarose gel. PCR performed on December 19, 2019, on extracted DNA from bone marrow showed that *Leishmania infantum* was the responsible species.

## 7. Patient Treatment and Follow-Up

Therapeutically, an isorhesis isogroup, blood transfusion constituting platelet concentrate, red blood cells, and fresh frozen plasma were administered on an emergency basis. For the management of the clinical symptoms, the patient was treated with ceftriaxone (100 mg/kg/day) combined with amikacin (15 mg/kg/day) for 15 days. Despite this clinical treatment, our patient still had episodes of external bleeding requiring regular blood transfusions. Therefore, in view of the urgency referring on clinical symptoms, we started treatment with meglumine antimoniate (Glucantime®) at 20 mg/kg/day. This treatment was administered in progressive doses for one week until the maximum dose was reached. The treatment was continued after confirmation of VL by PCR for 4 weeks with close biological and clinical follow-up. The result was highly effective from the day after treatment with cessation of the haemorrhagic syndrome, normalization of the haemodynamic state, improvement of respiratory distress, and stable apyrexia. Biologically, the control blood count showed a normalization of the white blood cell count, an increase in haemoglobin to 11.7 g/dl, but the persistence of thrombocytopenia at 4000 elements/mm^3^. In addition, the radiology control had shown a regression of pneumonia.

After 4 weeks of treatment with Glucantime® in hospitalization, the patient was discharged. During a control visit on January 14, 2020, regression of the adenopathy (to about 2 cm), disappearance of bleeding, and the infectious syndrome was noted. However, the splenomegaly had remained at the same stage.

On January 31, 2020, at the 8th week of treatment with Glucantime®, the patient was again admitted to the hospital due to the reappearance of fever and haemorrhagic syndrome associated with clinical anaemia. He still had type IV splenomegaly but no adenopathy or hepatomegaly. The blood count showed thrombocytopenia at 9000/mm^3^ and microcytic hypochromic anaemia at 8.4 g/dl with a normal white blood cell count. The C-reactive protein (CRP) measurement was positive at 68.4 g/dl. In view of this relapse of the clinical symptoms and the persistence of fever and thrombocytopenia, the failure of treatment with meglumine antimoniate might be the reason, and the replacement of this drug by amphotericin B in its liposomal form (Ambisome®) was considered. However, due to the unavailability of this drug in Senegal, a second course of Glucantime® was administered for 15 days with outpatient follow-up. However, after a two-month follow-up, the child was clinically stable with no adverse effects related to the treatment.

## 8. Comments of the Main Findings

Our observation showed that clinically our patient presented a tumor syndrome with large splenomegaly, adenopathies, and microcyte hypochromic anaemia and especially a recurrent haemorrhagic syndrome. Faced with this haematological emergency suggestive of leukaemia, it becomes important to integrate the diagnosis of VL. Indeed, the complexity of the picture of this disease has led to misdiagnosis that could be fatal to patients before confirmation. Hence, it is important to provide emergency treatment when major common haematological and infectious conditions have been eliminated.

Failure to adopt this strategy could be explained by the rarity of VL cases in Senegal and the limited routine molecular diagnostic tools in Senegalese hospitals.

A molecular diagnosis confirmed that our patient was infected by *L. infantum*. The 33% human seroprevalence of VL noted in Mont-Rolland showed frequent contact between human and the parasite [4]. Similarly, different studies have shown that in industrialized and developing countries, where *L. infantum* transmission occurred, cases of human VL were observed [8]. For the treatment, recommended drugs are liposomal amphotericin B and antimony derivatives. The choice between these drugs must take into account the clinical form of leishmaniasis, the terrain, the causal species, the availability and tolerance of the drugs, and their cost, especially in countries with limited resources [9]. Given the unavailability of amphotericin B, which is the first-line treatment, we used meglumine antimoniate (Glucantime®) for one month with ambulatory follow-up. Our experience shows that this drug is well tolerated and gave a spectacular regression of the main symptoms; however, it does not allow a radical treatment, as we had observed a relapse; between the 2 cures, we did not have a regression of splenomegaly symptoms. Therefore, there is an urgent need of amphotericin B in our care facilities for proper management of visceral leishmaniasis cases.

## 9. Conclusion

Our study highlighted a visceral leishmaniasis diagnosis in a 4-year-old child in the suburbs of Dakar in Pikine Teaching Hospital. The process leading to this result has shown the necessity to integrate this disease in the classical diagnostic approach in front of any clinical picture with a haemorrhagic, infectious, or tumoral syndrome after having eliminated the main common haematological and infectious disease symptoms. In this dynamic, it is important that our healthcare facilities have access to liposomal amphotericin B for the correct management of visceral leishmaniasis in Senegal. However, one of the most effective strategies for this zoonosis control remains preventive through early detection and treatment of patients and the eradication of vectors and the parasite reservoir (dogs).
